# Assessment of the Effect of Deleting the African Swine Fever Virus Gene *R298L* on Virus Replication and Virulence of the Georgia2010 Isolate

**DOI:** 10.3390/v16121911

**Published:** 2024-12-13

**Authors:** Elizabeth Ramirez-Medina, Lauro Velazquez-Salinas, Alyssa Valladares, Ediane Silva, Leeanna Burton, Douglas P. Gladue, Manuel V. Borca

**Affiliations:** 1 Foreign Animal Disease Research Unit, Plum Island Animal Disease Center (PIADC), Agricultural Research Service, U.S. Department of Agriculture, P.O. Box 848, Greenport, NY 11944, USA; lauro.velazquez@usda.gov (L.V.-S.); alyssa.valladares@usda.gov (A.V.); ediane.silva@usda.gov (E.S.); leeanna.burton@usda.gov (L.B.); 2 Foreign Animal Disease Research Unit, National Bio and Agro-Defense Facility, Agricultural Research Service, U.S. Department of Agriculture, Manhattan, KS 66502, USA; 3 Oak Ridge Institute for Science and Education (ORISE), Oak Ridge, TN 37830, USA

**Keywords:** ASFV, ASF, African swine fever virus, recombinant virus, ASFV virulence, ASFV *R298L* gene

## Abstract

African swine fever (ASF) is a lethal disease of domestic pigs that is currently challenging swine production in large areas of Eurasia. The causative agent, ASF virus (ASFV), is a large, double-stranded and structurally complex virus. The ASFV genome encodes for more than 160 proteins; however, the functions of most of these proteins are still in the process of being characterized. The ASF gene *R298L*, which has previously been characterized as able to encode a functional serine protein kinase, is expressed late in the virus infection cycle and may be part of the virus particle. There is no description of the importance of the *R298L* gene in basic virus functions such as replication or virulence in the natural host. Based on its evolution, it is proposed that there are four different phenotypes of *R298L* of ASFV in nature, which may have potential implications for *R298L* functionality. We report here that a recombinant virus lacking the *R298L* gene in the Georgia 2010 isolate, ASFV-G-∆R298L, does not exhibit significant changes in its replication in primary cultures of swine macrophages. In addition, when experimentally inoculated in pigs, ASFV-G-∆R298L induced a fatal form of the disease similar to that caused by the parental virulent ASFV-G. Therefore, deletion of *R298L* does not significantly affect virus replication and virulence in domestic pigs of the ASFV Georgia 2010 isolate.

## 1. Introduction

African swine fever (ASF) is a contagious disease of domestic and wild swine that can exhibit different clinical presentations depending on the virus isolate involved and the characteristics of the infected host [1]. ASF is currently distributed across Asia and Europe and has been recently detected in the Dominican Republic and Haiti [1]. Since 2007, ASF has significantly affected pig production and caused food shortages worldwide [1].

The causative agent of ASF, the ASF virus (ASFV), is a large and structurally complex double enveloped virus. The internal membrane surrounds a protein capsid that harbors a large double-stranded DNA genome of approximately 180–190 kilobase pairs capable of encoding more than 150 genes [2]. Many of these viral genes have not been experimentally characterized, and thus their function remains unknown, especially their role in virus functions such as replication and disease production in domestic swine. The understanding of gene function in ASFV has drastically improved with the development of recombinant viruses containing the deletion of the gene/s under study. This approach has been frequently used to evaluate gene function in the context of virus/cell interactions [3,4,5,6,7]. In addition, the identification of ASFV genes involved in virus virulence in domestic pigs, using such recombinant viruses, constituted an important primary stage in rational development of live attenuated ASFV vaccine candidates [8,9,10,11,12].

One of the genes that has not been studied in the context of virus infection either in vitro or in vivo is *R298L*. ASFV *R298L* encodes a functional serine protein kinase that is expressed late in the virus infection cycle and may be part of the virus particle [13]. It has been demonstrated that ASFV DNA synthesis in the nuclei of G0 cells is preceded by the activation of the viral genes *K196R*, *A240L*, *E165R*, *F334L*, *F778R*, and *R298L*, which are believed to be involved in the synthesis of nucleotides and the regulation of the cell cycle [14]. Therefore, it is possible that the ASFV *R298L* gene may be important in several of the virus functions.

Here we report that a recombinant virus harboring a deletion of the *R289L* gene, derived from the genome of the highly virulent Georgia isolate (ASFV-G), ASFV-G-∆R298L, showed the same replication ability in primary swine macrophage cultures as the parental virus. In addition, ASFV-G-∆R298L showed an almost indistinguishable virulent phenotype from that shown by ASFV-G. Therefore, under the conditions evaluated in this report, the *R298L* gene does not appear to play a significant role in ASFV replication or disease production in domestic pigs.

## 2. Materials and Methods

### 2.1. Viruses and Cells

Primary cultures of swine macrophages, at a density of 5 × 10^6^ cells per ml, were prepared as previously described [15]. ASFV Georgia (ASFV-G) is a virulent field strain kindly provided by Dr. Nino Vepkhvadze, from the Laboratory of the Ministry of Agriculture (LMA) in Tbilisi, Republic of Georgia [15]. Comparative growth curves between ASFV-G-∆R298L and the parental ASFV-G were conducted in primary swine macrophage cell cultures using a multiplicity of infection (MOI) of 0.01 HAD_50_ (hemadsorbing doses, as previously determined in primary swine macrophage cell cultures) [4]. Sample points were taken at 2, 24, 48, 72, 96, and 120 hours post infection (hpi), cells were frozen at ≤−70 °C, thawed, and the cell lysates titrated using primary swine macrophages in 96-well plates. The presence of virus infected cells was assessed by hemadsorption (HA) and the virus titers were calculated as previously described [16].

### 2.2. Development of the ASFV R298L Gene Deletion Mutant

ASFV-G-∆R298L, a recombinant ASFV with deletion of the ASFV gene *R298L*, was produced by homologous recombination using a recombination transfer vector (named p72mCherryΔR298L) as previously described [17]. The recombination vector p72mCherryΔR298L contains the ASFV-G genomic regions flanking the *R298L* gene: the left arm extends for approximately 1000 base pairs toward the left of nucleotide position 157,060 while the right arm extends approximately 1000 base pairs toward the right from nucleotide position 157,923, along with the reporter gene cassette including the mCherry fluorescent protein (mCherry) gene under the control of the ASFV p72 late gene promoter [18]. p72mCherryΔR298L was developed by DNA synthesis (Epoch Life Sciences, Sugar Land, TX, USA). Recombinant ASFV-G-∆R298L harbors a complete deletion of the *R298L* gene (between nucleotide positions 157,066–157,922). ASFV-G-∆R298L was purified by several limiting dilution steps based on mCherry activity detection and the recombinant virus stock full-length genome was sequenced using next-generation sequencing (NGS) following exactly the procedures previously described [3,4,5,6].

### 2.3. Detection of R298L Gene Transcription Kinetics

Transcription kinetics of the *R298L* gene were evaluated using a real-time PCR assay (qPCR) during the infection of ASFV-G in porcine macrophage cultures as previously described [18]. The expression of the early *CP204L* (p30) and late *B646L* (p72) ASFV genes was used as the reference. Porcine macrophage cultures were infected at an MOI of 10 with ASFV-G. RNA extraction was performed at 0–8 and 24 hpi using an RNeasy Kit (QIAGEN, Hilden, Germany). Extracted material was treated with 2 units of DNase I (BioLabs, San Diego, CA, USA) and then purified using the Monarch^®^ RNA Cleanup Kit (New England BioLabs, Inc., Ipswich, MA, USA). One ug of RNA was used to produce cDNA using qScript cDNA SuperMix (Quanta bio, Beverly, MA, USA), and then it was used for the qPCR. Primers and probes for the detection of the *R298L* gene were designed using the genome of ASFV Georgia 2007/1 strain (GenBank Accession #NC_044959.2). Primer forward: 5′-CCCGTCTCTGAAATGTTCTCG-3′, reverse: 5′-ACCAGCTTCCTTTAACCGTG-3′ and probe: 5′-FAM/TTAACCGCGACCATACCTATCGTCCA/MGBNFQ-3′. Primers and probes for the detection of *B646L* (p72) gene: forward 5′-CTTCGGCGAGCGCTTTATCAC-3′, reverse: 5′-GGAAATTCATTCACCAAATCCTT-3′ and probe: 5′-FAM-CGATGCAAGCTTTAT-MGB NFQ-3′. Primers and probes for the detection of *CP204L* (p30) gene: forward 5′-GACGGAATCCTCAGCATCTTC-3′, reverse: 5′-CAGCTTGGAGTCTTTAGGTACC-3′ and probe: 5′-FAM-TGTTTGAGCAAGAGCCCTCATCGG-MGB NFQ-3′. Primers and probes for the detection of the β-actin gene: forward 5′-GACCTGACCGACTACCTCATG-3′, reverse: 5′-TCTCCTTGATGTCCCGCAC-3′ and probe: 5′-FAM-CTACAGCTTCACCACCACGGC NFQ-3′. All qPCRs were conducted using the TaqMan Universal PCR Master Mix (Applied Biosystems, Wakefield, RI, USA) using the following amplification conditions: one step at 55 °C for 2 min, followed by one denaturation step at 95 °C for 10 min, then 40 cycles of denaturation at 95 °C for 15 s and annealing/extension at 65 °C for 1 min. Levels of transcription of different genes were quantified as relative quantities of mRNA accumulation (estimated by 2^−ΔΔCt^). The β-actin gene was used to normalize the transcription levels of different ASFV genes. Based on a previous validation in our laboratory, it was determined that values ≤ 18.44 2^−ΔΔCt^ were due to DNA background.

### 2.4. Evaluation of ASFV-G-ΔR298LR Virulence in Domestic Pigs

The virulence of ASFV-G-ΔR298L was assessed in 35–40 kg commercial breed swine. Groups of 5 pigs were intramuscularly (IM) inoculated with 10^2^ HAD_50_ of ASFV-G-∆R298L and compared with a group of animals inoculated with 10^2^ HAD_50_ of the virulent parental virus ASFV-G. The presence of clinical signs associated with ASF (such as anorexia, depression, fever, purple skin discoloration, staggering gait, diarrhea, and cough) as well as body temperature values were recorded daily throughout the experiment. Experiments with pigs were performed under biosafety level 3 conditions in the animal facilities at Plum Island Animal Disease Center, following a strict protocol approved by the Institutional Animal Care and Use Committee (225.06-19-R_090716, approved on 09-06-19).

## 3. Results and Discussion

### 3.1. Evolutionary Dynamics of R298L Gene of ASFV in Nature

**Figure 1:**
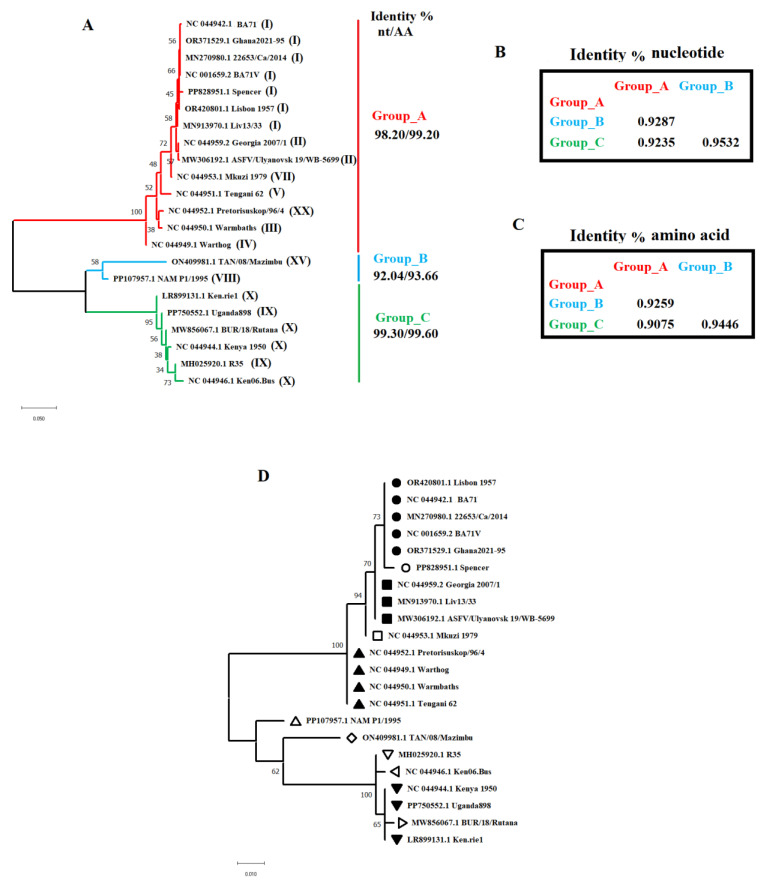
Phylogenetic dynamics of *R298L* gene in nature. (**A**) Phylogenetic analysis conducted by the neighbor-joining method using the full-length sequence of the *R298L* gene indicates the existence of three potential phylogenetic groups. Numbers in the parenthesis indicate the genotype of different strains based on the *B646L* classification. Percentage of nucleotide (nt) and amino acid (AA) identities within groups are displayed. Pairwise distance analysis showing differences at the nucleotide (**B**) and amino acid level (**C**) between phylogenetic groups are exhibited. (**D**) Phylogenic tree reconstructed by the maximum likelihood method using full length amino acid sequences of *R298L*. ASFV labeled with the same shape indicates 100% amino acid sequence identity.

The ASFV *R298L* gene encodes a serine protein kinase in ASFV [13]. To obtain more insights about the evolutionary dynamics of this gene in nature, we conducted a BLAST [19] analysis using the *R298L* gene of the isolate Georgia 2007/1 as the query. As a result, a total of 22 representative sequences were retrieved from the GenBank database. These sequences included ASFV lineages I, II, III, IV, V, VII, VIII, IX, X, XV and XX (Figure 1). Phylogenetic analyses inferred using the neighbor-joining method and the maximum composite likelihood method as a substitution model indicated that the *R298L* gene of ASFV has diverged in at least three distinct phylogenetic groups in nature (Figure 1A). The Georgia 2007/1 isolate used in this study as a parental strain to produce the recombinant virus ASFV-G-ΔR298L was associated with the conserved phylogenetic group A, sharing high levels of nucleotide and amino acid identities with isolates associated with the other six ASFV lineages (Figure 1A). Overall, pairwise distance conducted among representative isolates reflected the high conservation of this gene in nature (0.956 ± 0.004). When we conducted this analysis between phylogenetic groups, we found that the greatest distance at both the nucleotide (Figure 1B) and amino acid (Figure 1C) levels was between group A and the other two groups, suggesting that *R298L* in nature might be evolving into different isoforms.

In terms of the evolution of *R298L* within lineage II, pairwise distance analysis carried out between the two representative strains associated with this lineage showed extremely high conservation of this gene during the evolution of this lineage in nature (nucleotide: 0.998 ± 0.010 and amino acid: 1.000). The only change during the evolution of this lineage has implicated the synonymous substitution GCG-GCA at codon 20, indicating that *R298L* is not promoting the divergence of lineage II during pandemics. To identify potential isoforms of *R298L* in nature and how these are related at the phylogenetic level, we conducted a phylogenetic analysis using the maximum likelihood method and JTT matrix-based model using the amino acid sequences of the representative isolates. Overall, a total of 12 possible distinct isoforms of *R298L* were found distributed among the different phylogenetic groups (Figure 1D). Interestingly, the amino acid sequence of the representative isolates linked with lineage II (Geogia 2007/1 and ASFV/Ulyanovsk 19/WB-5699) appeared 100% identical to the sequence displayed by the strain Liv13/33 (Genotype I), isolated in 1983 from a tick in Zambia, Africa [20]. The statement above suggests that the protein sequence of *R298L* present in the lineage II has remained unchanged for at least 40 years.

**Figure 2:**
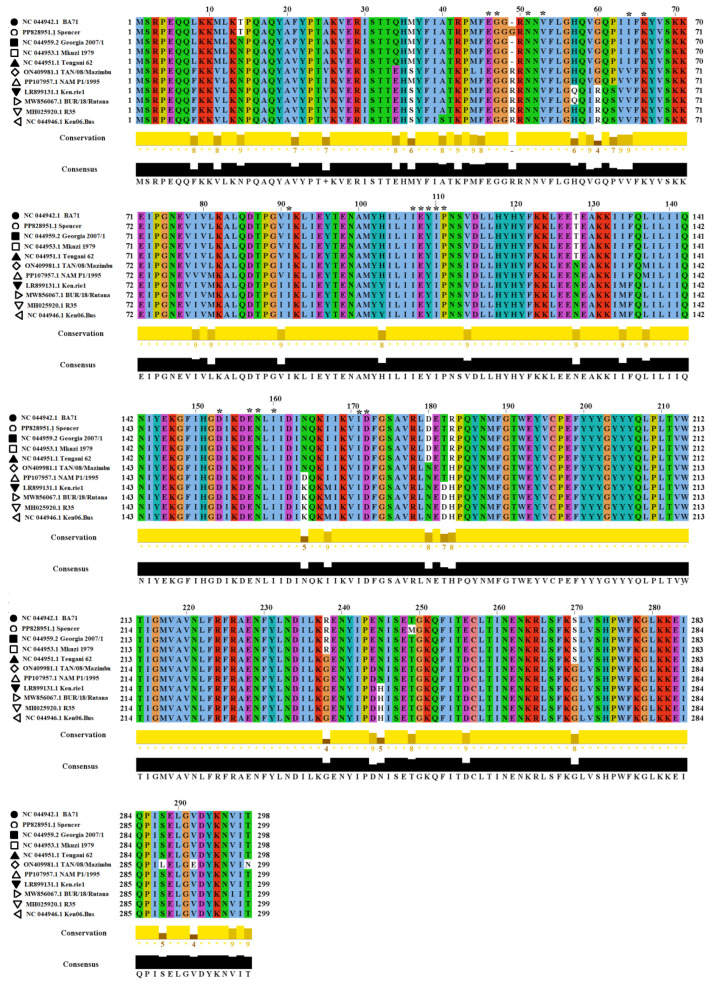
Amino acid similarities of *R298L* among ASFV representative strains. Amino acid alignment showing similarities of the *R298L* protein among a group of representative ASFV isolates. The protein kinase/catalytic domain spans between residues 46 and 277. Asterisks above specific sites indicate ATP binding residues in *R298L*. Conservation plot scores reflect the nature of the change in specific sites. Increased scores reflect substitutions between residues with similar biological properties. The analysis was conducted in the software Jalview version 2.11.1.7.

Overall, the amino acid aligment of *R298L* showed the high conservation of this protein among ASFV strains in nature. A total of 40 variable sites were predicted in the aligment (Figure 2). From these, site 49 is characterized by the presence of an insertion (arginine/R), which is characteristic of the isolates comprising the phylogenetic groups B and C. A different insertion (glycine/G) at this site was also found in a single isolate of the phylogentic group A (spencer). Interestingly, these insertions are located in the the protein kinase/catalytic domain (spanning between residues 46 and 277), suggesting that the presence of these insertions could have a possible effect in the funcionality of this domine. In general, there is high conservation among the 18 residues associated with the ATP binding sites (Figure 2). The only exception is residue 64, which represents a variable site, showing an isoleucine/I in isolates related to phylogenetic group A, while isolates from groups B and C are associated with the presence of a valine/V (Figure 2). In this sense, not only the presence of the insertions but also the differences at ATP binding site 64 may be proposed as a potential markers for diagnostic or epidemiologic purposes. Future experimental studies are needed to evaluate the possible biological significance of insertions and differences at binding site 64 found in *R298L*.

### 3.2. Effect of Natural Selection in the Evolution of R298L

**Figure 3:**
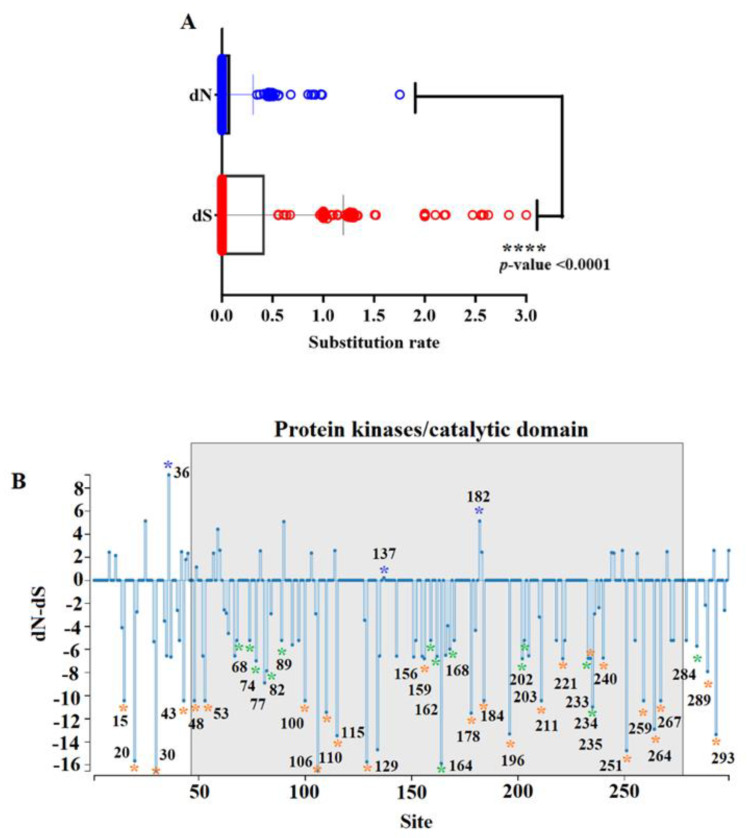
Evolutionary dynamics of *R298L* gene in nature. (**A**) Comparison between synonymous (dS) and nonsynonymous (dN) substitutions rates during the evolution of the *R298L* gene. Significant differences between dS and dN were determined by the unpaired *t*-test. (**B**) Graphic representation obtained by SLAC analysis, showing the ratio dN-dS at specific codon sites in the *R298L* gene of ASFV. Identification of specific codon sites under positive selection (blue asterisks) and negative selection (green asterisks). Orange asterisks represent sites of negative selection at internal nodes. Results were obtained by MEME (*p*-value threshold of 0.1) and FEL (*p*-value threshold of 0.1). Numbers close to the asterisk indicate the specific codon position.

To identify critical codon sites during the evolution of *R298L*, we conducted multiple selection detection methods, including single likelihood ancestor counting (SLAC) [21], fixed effects likelihood (FEL) [21], and mixed effects model of evolution (MEME) [22]. These methods detect codon sites under diversifying (selection favoring the fixation of nonsynonymous changes) or negative selection (selection against the fixation of nonsynonymous changes) by inferring rates of synonymous (dS) and nonsynonymous (dN) substitutions on a per-site basis in a codon-based phylogenetic framework [23]. An overall dN/dS rate equal to 0.189 indicates that negative selection is dominating the evolution of *R298L* in nature. This result is consistent with the increased substitution rate at synonymous codon sites, which was calculated to be 5.11 times higher than the one observed at nonsynonymous sites (Figure 3A). Overall, a total of 39 codon sites along the *R298L* gene were identified as being under negative selection by FEL (Figure 3B). Most of these codons (82.05%) are located in the protein kinase/catalytic domain. Interestingly, when we conducted FEL analysis at internal nodes, we found strong evidence of negative selection at the population level in 25 out of 39 codons previously predicted (Figure 3B), highlighting the potential relevance of these codon sites for the functionality of *R298L*. Among these sites, we found evidence of negative selection at the population level at two codons associated with highly conserved ATP binding residues (see the alignment in Figure 2): V53 (alleles in the population GTA, GTC, and GTT), and I110 (alleles in the population ATT and ATC), stressing the importance of the conservation of these residues during the evolution of *R298L* of ASFV. Furthermore, considering the time of collection of some of the representative ASFVs included in these analyses, like Kenya 1950 (Figure 1), we may state that the conservation of the amino acid residues linked to the 39 codon sites identified under negative selection at *R298L* have remained in the same state for at least 70 years, emphasizing their possible relevance in maintaining the functionality of *R298L* during its evolution in nature.

Overall, based on the strong evidence of negative selection at the population level, we consider that the codons identified during our study represent an excellent framework for future experimental studies attempting to identify new functional sites in *R298L*. We used MEME to identify codon sites under positive selection. As a result, three codons associated with the encoded residues 36, 137 and 182 were found to promote the adaptation of *R298L* in nature; furthermore, 137 and 182 are part of the protein kinase/catalytic domain (Figure 3B).

**Figure 4:**
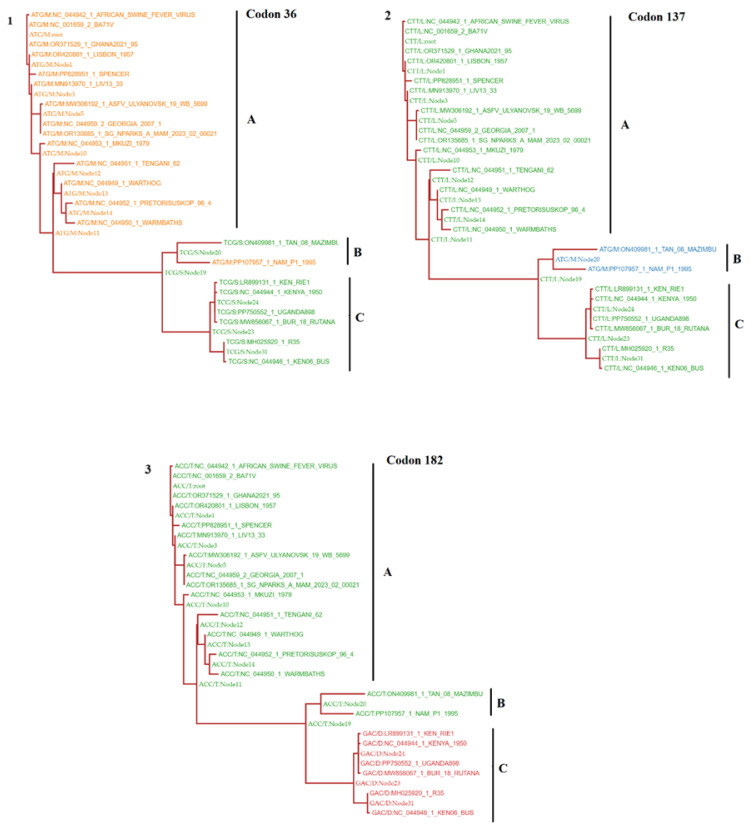
Ancestral reconstruction analysis of codon sites under positive selection at *R298L*. The analysis shows the evolutionary dynamics among the predicted phylogenetic groups in *R298L* at codon 36 (**A**), 137 (**B**) and 182 (**C**). Each phylogenetic tree displays the predicted codon sequences at internal nodes (most probable common ancestor sequence associated with the divergence between and within phylogenetic groups) and leaf nodes (represented by different isolates). Analysis was conducted using the algorithm MEME. Results were saved in json format and visualized in the MEME analysis result visualization tool (https://observablehq.com/@spond/meme, accessed on 25 October 2024).

To get more insights about the impact of the selection of these codons in the divergence of different phylogenetic groups using SLAC, we conducted an ancestral reconstruction analysis (Figure 4). Overall, based on the selection of codon 36, we observed that representative isolates can be divided into two different phenotypes: M36 and S36 (Figure 4-1).

The allele ATG(M) appeared highly conserved among ancestral predicted sequences at internal nodes and representative ASFV linked to group A. Conversely, allele TCG(S) was representative of ancestral states and ASFV associated with groups B and C. However, the only exception was the strain NAM/P1/1995, collected from a pig in Namibia [24] (related to phylogenetic group B), which evolved with selection of the allele ATG(M), suggesting that convergent/parallel evolution is implicated in the presentation of the phenotype M36 in nature. On the other hand, selection of codons 137 and 182 were implicated in the divergence of phylogenetic groups B and C, respectively (Figure 4-2,3). Interestingly, when we conducted FEL at internal nodes, we found evidence of positive selection at the population level (*p* ≤ 0.1) in codon 182, stressing the relevance of this codon during the adaptation of *R298L* in nature. Experimental analyses are needed to get more insight about the potential role of the codons identified under positive selection in the functionality of *R298L*. Based on the results obtained by MEME, we may propose the existence of four different phenotypes of *R298L* of ASFV in nature. Phenotype 1: M36-L137-T182 (Group A), phenotype 2: S36-M137-T182 (Group B), phenotype 3: M36-M137-T182 (Group B), and phenotype 4: S36-L137-D182 (Group C). In light of these results, we may state that the identification of these phenotypes may have two potential implications. First, at the epidemiologic level to support the characterization of the genetic origin of ASFV, linked to specific phylogenetic groups based on the *R298L* classification proposed in this study. Second, the potential existence of different phenotypes may imply that *R298L* proteins present in ASFV at different phylogenetic groups might show differences in their functionality, potentially impacting their pathogenesis in pigs. Future experimental studies are needed to evaluate this hypothesis.

### 3.3. Effects of Epistasis/Co-Evolution at R298L

**Figure 5:**
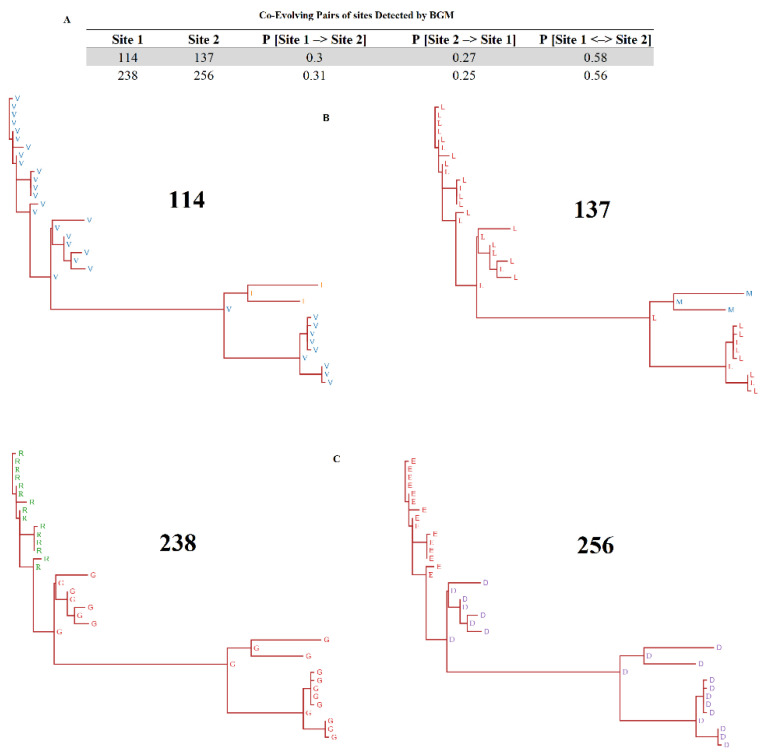
Detection of epistasis/co-evolution in *R298L*. (**A**) Pairs of co-evolving sites identified by BMG analysis (posterior probability cutoff 0.5). P [Site 1 –> Site 2] indicates the probability of site 2 to be conditionally dependent on site 1. P [Site 2 –> Site 1] indicates the probability of site 1 to be conditionally dependent on site 2. P [Site 1 <–> Site 2] indicates the probability that sites 1 and 2 are not conditionally independent. Ancestral reconstruction analysis using MEME was conducted to show the evolutionary dynamics of pairs of co-evolving sites 114–137 (**B**) and 238–256 (**C**). For each phylogenetic tree, the predicted amino acid sequences at internal nodes are shown (most probable common ancestor sequence associated with the divergence between and within phylogenetic groups) and leaf nodes (represented by different isolates). Phylogenetic trees were obtained using the MEME analysis result visualization tool (https://observablehq.com/@spond/meme, accessed on 25 October 2024).

Furthermore, to identify evidence of epistasis/co-evolution in *R298L*, we conducted the SpiderMonkey-Bayesian Graphical Model (BGM) [25]. BGM is deduced from reconstructed substitutions at each branch/site combination to infer conditional evolutionary dependencies of sites in the alignments, denoting interactions among variable residues in proteins [25]. BMG found evidence of two pairs of co-evolving sites (posterior probability > 0.5) at *R298L* (Figure 5A). All four sites constituting the two pairs of co-evolving sites are located in the protein kinase/catalytic domain, highlighting the relevance of these interactions. Interestingly, we found that positive selection at codon 137 was influenced by the evolution of site 114 (Figure 5B). Not only all representative ASFVs, but also the predicted sequences at ancestral nodes, showed the phenotypes V114-L137 and I114-M137 consistently, stressing the relevance of this result at population level. Similar results were observed in the second pair of co-evolving sites (Figure 5C). There, the phenotypes R238-E256 and G238-D256 were observed. The above data strongly suggest that epistasis should be considered as an evolutionary mechanism of *R298L* of ASFV in nature. No evidence of recombination was found in *R298L* after conducting the genetic algorithm for recombination detection [26].

### 3.4. Transcription of the R298L Gene in ASFV Infected Macrophages

**Figure 6:**
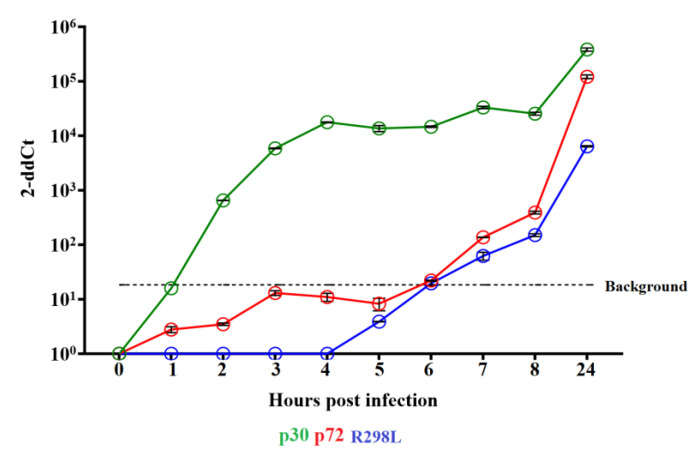
Expression of ASFV gene *R298L* in swine macrophages infected with ASFV-G. Reverse transcription qPCR was performed to assess the expression of the *R298L* gene at different time points post infection. Results obtained using specific qPCRs to detect the expression of ASFV genes encoding early protein p30 and late protein p72 are used as references. Transcription levels of different ASFV genes are expressed as relative quantities of mRNA accumulation (estimated by 2^−ΔΔCt^). Values are expressed in log_10_. Dashed line reflects the background (≤1.26 log_10_ 2^−ΔΔCt^) associated with potential traces of DNA contamination after treatment of the RNA samples with DNase I.

The transcription kinetics of the *R298L* gene was evaluated during the virus replication cycle in swine macrophages infected with ASFV strain Georgia. Swine macrophage cultures were infected (MOI = 10) with ASFV-G and cell lysates were obtained from 0–8 and 24 hpi. The presence of *R298L* transcripts was evaluated by RT-PCR. As a reference, we used the well-characterized ASFV early protein p30 (*CP204L* gene) and the late protein p72 (*B646L* gene). Overall, similar transcriptional kinetics were observed between *R298L* and p72 (*B646L*), indicating that *R298L* is a late transcribed gene (Figure 6). In both genes, low initial transcription levels were observed at 7 hpi and then they gradually increased until the end of the experment. These results are consistent with a previous report that indicated that *R298L* is classified as a low to mid transcribed gene [13].

### 3.5. Development of the Recombinant ASFV-G-ΔR298L

Since ASFV gene *R298L* encodes for a functional serine protein kinase that may be part of the virus particle and has been proposed to be involved in virus DNA synthesis [13], it could be assumed that this gene may play an important role in processes such as virus replication or virulence in domestic pigs.

**Figure 7:**
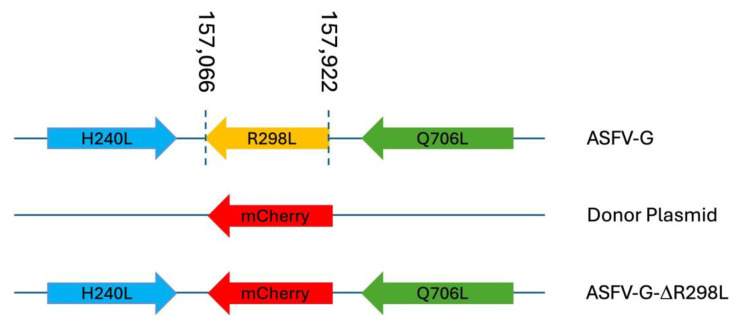
Schematic for the development of ASFV-G-∆R298L. The recombinant vector, containing the mCherry reporter gene under the ASFV p72 promoter activity and the gene positions are shown. The nucleotide positions of the area that was deleted in the ASFV-G genome are indicated by the dashed lines. The resulting ASFV-G-∆R298L virus with the cassette inserted is shown on the bottom.

To understand the potential role of the *R298L* gene in these processes, a recombinant virus was developed harboring the deletion of the *R298L* gene (ASFV-G-∆R298L) using as parental virus the highly virulent ASFV Georgia 2010 isolate (ASFV-G). The *R298L* gene was completely deleted and then substituted by the p72mCherry cassette [18]. An area covering 687 bp (involving nucleotide positions 157,066 and 157,922) was deleted from the ASFV-G genome and substituted with a 1226-bp cassette containing the p72mCherry construct (see Section 2) (Figure 7). The recombinant ASFV-G-∆R298L stock was purified by limiting dilution, using primary swine macrophage cell cultures as the cell substrate.

The nature of the modifications introduced into the ASFV-G-∆R298L genome was assessed by analyzing the full genome sequence by NGS using an Illumina NextSeq^®^ 500. The results of the analysis indicated a deletion of 861 nucleotides and an insertion of 1226 nucleotides corresponding to the p72-mCherry cassette sequence. There were no undesired additional genomic modifications detected in the stock of ASFV-G-∆R298L. NGS revealed no evidence of the potential contamination of the parental ASFV-G genome into the ASFV-G-∆R298L stock.

### 3.6. Replication of ASFV-G-∆R298L in Primary Swine Macrophages

**Figure 8:**
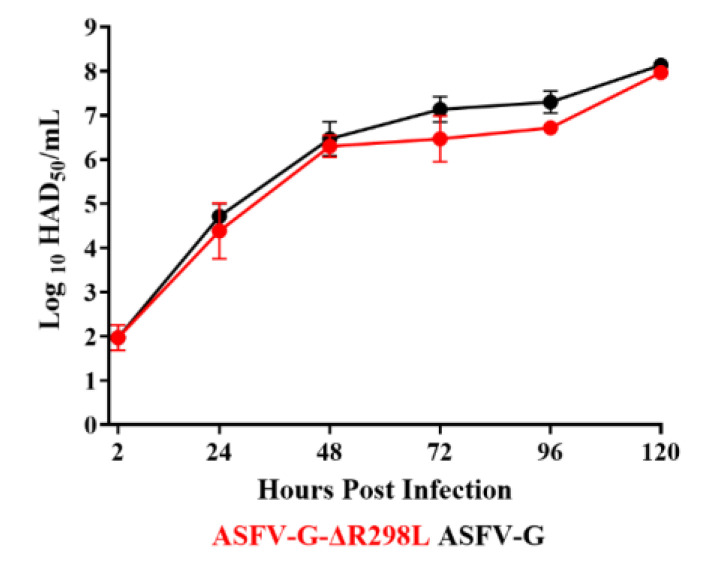
In vitro growth kinetics in primary swine macrophage cell cultures for ASFV-G-∆R298L and parental ASFV-G (MOI = 0.01). Data represent means and standard deviations of two replicas. Sensitivity using this methodology for detecting virus is ≥log10^1.8^ HAD_50_/mL.

To evaluate the importance of the *R298L* gene during the process of virus replication in swine macrophages, the replication ability of ASFV-G-∆R298L in swine macrophage cultures was studied and compared to that of the parental ASFV-G. A multistep growth curve study was performed, infecting swine macrophage cultures with an MOI of 0.01 with either ASFV-G-∆R298L or ASFV-G and virus yields were quantified at 2, 24, 48, 72, 96, and 120 hpi. The results demonstrated that ASFV-G-∆R298L replicates as efficiently as the parental ASFV-G does with similar virus titers at all of the sample points tested (Figure 8), indicating that deletion of the *R298L* gene from the genome of the ASFV-G does not significantly impact the ability of the virus to replicate in swine macrophage cultures.

### 3.7. Assessment of ASFV-G-∆R298L Virulence in Swine

To evaluate the potential role of the *R298L* gene in the production of disease by the ASFV-G isolate, the recombinant ASFV-G-∆R298L was experimentally inoculated by the IM route into a group of five pigs at a dose of 10^2^ HAD_50_. A control group of four animals was inoculated under the same conditions with the virulent parental ASFV-G isolate. The appearance of clinical signs associated with ASF was observed in both groups of animals for a 28-day period.

**Figure 9:**
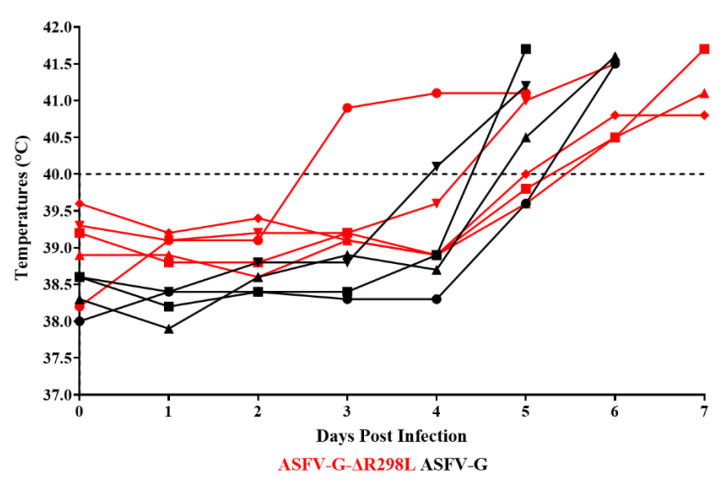
Evolution of body temperature in animals (5 animals/group) IM infected with 10^2^ HAD_50_ of either ASFV-G-∆R298L or parental ASFV-G.

**Figure 10:**
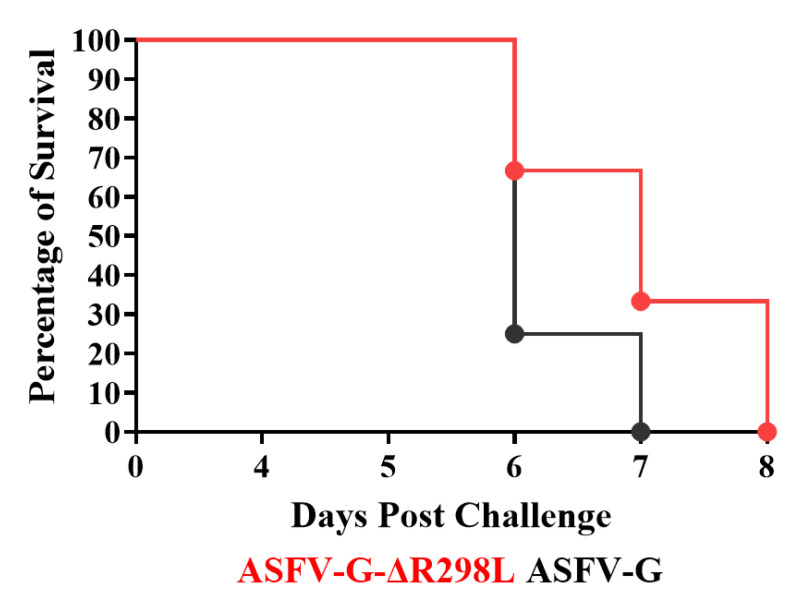
Evolution of mortality in animals IM infected with 10^2^ HAD_50_ of either ASFV-G-∆R298L or parental virulent ASFV-G.

Animals inoculated with the virulent parental ASFV-G showed an increase in body temperature (>40 °C) on day 4–5 pi and quickly progressed to a full clinical disease (depression, anorexia, staggering gait, diarrhea, and purple skin discoloration) (Figure 9 and Figure 10), with all pigs euthanized on day 5–6 post infection (pi).

Similarly, pigs IM inoculated with ASFV-G-∆R298L presented a lethal form of the disease like that in animals inoculated with ASFV-G. Animals showed increased body temperature over 40 °C (dash line indicates the top limit of normal body temperature) on days 4 to 6 pi with the clinical disease promptly worsening, with all animals being euthanized between days 6 and 8 post infection due to the severity of the clinical disease. Therefore, deletion of the *R298L* gene from the ASFV-G genome does not produce alterations in virus virulence when tested in experimentally infected domestic swine under the experimental conditions described here.

**Figure 11:**
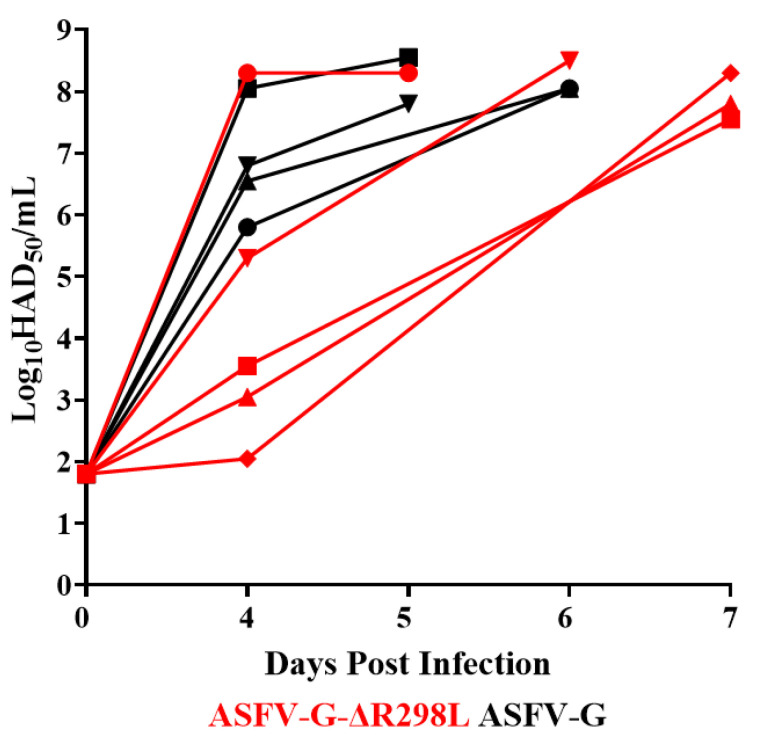
Viremia titers detected in pigs IM inoculated with 10^2^ HAD_50_ of either ASFV-G-∆R298L or ASFV-G. Each symbol represents individual viremia titers in each animal in the groups. Sensitivity of virus detection: ≥log10 ^1.8^ TCID_50_/mL.

The degree of replication of ASFV-G-∆R298L in the experimentally infected pigs was evaluated by measuring the levels of viremia in comparison to the viremia values shown in the pigs infected with the parental virulent ASFV-G (Figure 11). Pigs experimentally infected with ASFV-G showed high viremia titers (ranging from 10^5.55^–10^8^ HAD_50_/mL) at day 4 pi. High values (ranging from 10^7.55^–10^8.2^ HAD_50_/mL) lasted until day 5 or 6 pi when all animals were euthanized. Similarly, viremia titers in animals inoculated with ASFV-G-∆R298L also presented high viremia titers. These animals presented a wide range in titer values (between 10^2^–10^8.2^ HAD_50_/mL by day 4 pi, drastically increasing their viremias titers (ranging from 10^7^–10^8.2^ HAD_50_/mL) by day (5 to 7 pi), when all of them had to be euthanized due to the severity of the clinical disease.

It should be remarked that the genomic sequence of the virus obtained in blood samples taken from these five animals inoculated with ASFV-G-∆R298L confirmed that the virus present in each of the samples sequenced by NGS was ASFV-G-∆R298L. These results confirm that in these animals, the clinical disease was in fact induced by ASFV-G-∆R298L and not due to potential cross contamination with the parental ASFV-G not completely eliminated during the process of ASFV-G-∆R298L purification. In addition, no unexpected genomic modifications (mutations or genomic rearrangements) were observed in the genome of the viruses isolated from the ASFV-G-∆R298L inoculated animals. Therefore, ASFV-G-∆R298L developed a systemic infection in experimentally inoculated animals that resembles that induced by the parental ASFV-G.

The results reported here clearly show that deletion of the *R298L* gene from the genome of the highly virulent isolate Georgia 2010 does not affect basic ASFV functions such as virus replication in macrophages (both in cell cultures and pigs experimentally infected) or its virulence in domestic pigs. It is possible that other viral genes that have not been fully characterized yet may have similar or partially overlapping functions with the *R298L* gene. It is interesting to note this is another example where an ASFV gene, which was previously shown to have a defined structure and/or function that would presuppose a critical role in important functions of the virus, is shown not only to be non-essential for virus viability, but its absence also does not affect virus replication or disease production in the natural host. Similar situations have been observed with other ASFV genes initially demonstrated to have a specific function using experimental in vitro approaches [3,27,28,29,30,31,32,33,34,35,36,37,38,39]. This fact emphasizes the importance of evaluating the roles and functions of gene function in the context of the interaction between the virus and the host (either cell cultures and/or susceptible animals) rather than encoded proteins as isolated factors within in vitro models.

## Data Availability

All data are included in the manuscript.
